# Predictors of relapse of acute malnutrition following exit from community-based management program in Amhara region, Northwest Ethiopia: An unmatched case-control study

**DOI:** 10.1371/journal.pone.0231524

**Published:** 2020-04-22

**Authors:** Dereje Birhanu Abitew, Alemayehu Worku Yalew, Afework Mulugeta Bezabih, Alessandra N. Bazzano

**Affiliations:** 1 School of Public Health, Addis Ababa University, Addis Ababa, Ethiopia; 2 School of Public Health, Mekelle University, Mekele, Ethiopia; 3 Department of Global Community Health and Behavioral Sciences, School of Public Health and Tropical Medicine, Tulane University, New Orleans, LA, United States of America; University of the Witwatersrand, SOUTH AFRICA

## Abstract

**Background:**

Community-based management of acute malnutrition (CMAM) is an effective program to manage children with acute malnutrition, including both severe and moderate acute malnutrition. However, little is known about continued child nutritional status after discharge from community based management of acute malnutrition programs in Ethiopia.

**Objective:**

The study aimed to identify factors associated with relapse of acute malnutrition among children 6–59 months after been discharged recovered from community based management program in South Gondar Zone, Northwest Ethiopia.

**Methods:**

A case-control study was conducted in three districts of South Gondar Zone by tracing children age 6–59 months who were reported as recovered from the community based management program. Sample size calculated for the first objective of assessing prevalence of severe acute malnutrition among children following discharge as recovery using Epi- Info version 7.1.3.3 StatCalc taking 95% CL, 17.8% post discharge relapse (Ashraf H, et al. (2012), 3% margin of error, design effect of 2 and adding 5% non-response rate was the largest sample size and used to this study.

Children with Mid Upper Arm Circumference (MUAC) <12.5cm constituted cases and children with > = 12.5cm served as controls. Data were collected from 10 November 2017 to 30 January 2018 using a survey questionnaire and families were asked to bring children to a health facility for anthropometric measurements, following which data were entered and analyzed. Bivariate and multivariable logistic regression models were utilized to measure association between the risk factors and acute malnutrition.

**Results:**

Overall, 1,273 participants were interviewed. The mean age in months of children was 23.1 (±9.1 SD) for cases and 23.1 (±8.9 SD) for controls. About 40% of the cases and 50% of the controls were female children. The factors associated with acute malnutrition were: male children (AOR = 1.84, 95% CI: 1.42–2.39), living in a food insecure household (AOR = 1.67, 95% CI:1.15–2.44), non-receipt of Vitamin A supplement (AOR = 1.76, 95% CI: 1.28–2.41), prelacteal feeding (AOR = 2.81 (95%CI, 1.57–5.05), distance to water source more than 15 walk (AOR = 1.88, 95% CI:1.32–2.71), less frequent self-reported hand washing (AOR = 1.35, 95% CI:1.05–1.75), mother not having consumed extra food during this pregnancy/lactation (AOR = 1.36, 95% CI: 1.03–1.78), and respondent age above 30 years (AOR = 1.43, 95% CI:1.10–1.87).

**Conclusion:**

The key factors contributing to relapse of acute malnutrition were related to childcare and feeding practices. Social and behavior change communication strategies targeting families at risk of undernutrition, along with improved food security and integrated programming are recommended to prevent relapse of acute malnutrition.

## Introduction

### Background

Under-nutrition results from reductions in food intake or diet quality and is often combined with pathological causes [1, 2] and may be categorized as either chronic or acute malnutrition [3]. Acute malnutrition or wasting is further classified as moderate acute malnutrition (MAM) or severe acute malnutrition (SAM) based on the degree of malnutrition and the presence of edema [3, 4]. According to a 2018 report, globally over 49 million children under 5 have acute malnutrition and nearly 17 million were classified as severely wasted [5]. The magnitude of acute malnutrition nationally and in the Amhara region was found to be 10% [6]. It is not only the magnitude of undernutrition, but also subsequent health consequences that are important, as children suffering from acute malnutrition have weakened immunity and face an increased risk of death, particularly when wasting is severe [5]. The mortality rate among children with SAM is 5–20 times higher than among well-nourished children [3,7]. Globally about 1 million children die every year from SAM [2, 8] and in Ethiopia about 57% of all under-five deaths are related to malnutrition, of which three-quarters are related to mild to moderate malnutrition [9]. It is also estimated that about 70% of all childhood mortality in developing countries is due to five major conditions, and for these, malnutrition increases the likelihood of mortality up to 56% [2]. Ten high-impact, nutrition-specific interventions have been identified that, if taken as a package up to 90 percent coverage, could reduce wasting by 60 percent. Among these important interventions is the management of SAM and MAM [10]. Children with uncomplicated SAM (WHZ below -3 SD cut-off and/or with MUAC cut-off of 11.5 cm and/or with bilateral edema) and MAM (WHZ between −2 and −3 or mid-upper arm circumference (MUAC) between 11–5 and 12.5 centimeters) may be treated in the community setting with special therapeutic foods without requiring admission to a health facility, referred to as Community based Management of Acute Malnutrition (CMAM) program [11, 12]. The most common therapeutic regimens are based on severity and include a short course of basic oral medications to treat infections [3].

Children diagnosed with SAM are discharged recovered from the program when their weight-for-height/length is ≥–2 Z-score and they have had no oedema for at least 2 weeks or alternatively if their mid-upper-arm circumference is ≥12.5 cm and they have had no oedema for at least 2 weeks. In addition, children admitted with only bilateral pitting oedema are discharged recovered based on the anthropometric indicator routinely used in local programmes [4] which in the Ethiopian context, is when their W/L> = 85% or W/H> = 85% on more than one occasion (two days for in-patients, two weeks for out-patients) and they have had no oedema for 10 days (as an in-patient) or 14 days (as an out-patient) and/or, when target weight gain has been reached and they have had no oedema for 10 days (as an in-patient) or 14 days (as an out-patient) as the second option [13].

The CMAM program has been reported to be effective in terms of access and key performance indicators (recovery, default, and death rates) [7, 14]. In some research findings, the recovery rate has been reported to be above the Sphere Handbook [15] minimum standard of >75% [16–19], but the relapse rate of acute malnutrition (both SAM and MAM) after discharge as recovered is high. This has been noted as 78% as in Bangladesh **(**69% MAM & 9% SAM) [20], 27% (10% SAM & 17% MAM) [21], and in Southern Ethiopia 72.1% (34.6% SAM & 37.5% MAM) [22]. Some studies [21, 23–25] have investigated the factors specifically associated with relapse of acute malnutrition after been discharged recovered, but none thus far have emanated from Ethiopia as a whole, or the Amhara region in particular. The objective of the study was therefore to identify the factors related to relapse of acute malnutrition following successful recovery through community-based management of acute malnutrition (CMAM) program in the previous one year in Northwest Ethiopia.

## Materials and methods

### Study area

The study was conducted in South Gondar Zone of Amhara region, Ethiopia. South Gondar Zone is one of the 11 administrative zones of Amhara region, Ethiopia. Debretabor is the capital city of the zone situated about 100 Km East of Bahir Dar (the capital city of Amhara Region) and 667 Km North of Addis Ababa (the capital city of Ethiopia). The Zone has 17 districts, five of which are town administrations namely Debretabor, Woreta, Mekane Eyesus, Addis Zemen, and Nefas-Mewcha. According to the Government of Ethiopia, the 2017/18 population of the Zone was 2,484,929 of which 183, 525 were children 0-4yrs old [26]. In South Gondar, there was one Zonal hospital and three district hospitals, 90 Health Centers, 378 Health Posts, and more than 10 private clinics. According to the Zonal health department annual report, 68% of children under two years of age had participated in a growth monitoring program, and a total of 6,468 SAM children were managed in the health facilities with an overall 96.8% recovery rate [27]. Regarding to the topography of the randomly selected districts, Tach-Gayint is one of the highland and mountainous districts in the Zone next to Lay-Gayint, while Ebnat is lowland but has hilly places, however all three districts are drought-prone, rural and land degraded.

### Study design and population

An unmatched case-control study was conducted from 10 November 2017 to 30 January 2018. The source populations were 6–59 months old children discharge as recovered from CMAM program in South Gondar Zone and the study populations were those children age 6–59 months following discharge as recovered in the randomly selected district of South Gondar Zone, Amhara region, Ethiopia.

### Definition of cases and controls

Case and control children were identified from those discharged as recovered after their mid upper arm circumference (MUAC) was taken and edema was assessed.

Case: a child was considered as case, if he/she had MUAC <12.5cm (both acute and severe acute malnutrition) and/or presence of bilateral edema) after being successfully discharged recovered from CMAM program.

Control: a child was considered as control, if he/she has MUAC > = 12.5cm and/or with no bilateral edema after being successfully discharged recovered from CMAM program.

### Inclusion and exclusion criteria

#### Inclusion criteria

Children 6 to 59 months old who were discharged recovered from CMAM program.

#### Exclusion criteria

Children were excluded if their name was different from the name present in the CMAM registration logbook. In addition, children with chronic illnesses such as cerebral palsy or congenital malformations were also excluded.

*Sample size determination and sampling procedure*. Sample size was calculated using Epi-Info version 7.1.3.3 StatCalc with 95% CL, 80% power, 1:2 case to control ratio, with child vaccination status as the main exposure (19.3% incomplete vaccination status and AOR = 1.89) [24], a design effect of two, and considering 10% non-response rate. The calculated sample size for the study was 1,103 (368 cases and 735), but as this was part of a large study, the final sample size of 1290 was used to increase the precision, resulting in 430 cases and 860 controls.

Regarding the sampling procedure, a two stage sampling technique was used. Among the 17 districts of South Gondar Zone, 12 were rural administrative districts and from these rural districts, 3 districts (Ebnat, Tach-Gayint, and Lay- Gayint) were selected randomly using a lottery method within which, 10 health centers were selected randomly (129 discharged recovered children per health Centre). In the current Ethiopian health system, a health Centre has 5 cluster health posts in its catchment area and we considered 3–5 health posts per health Centre based on reported caseload, therefore 26–43 discharged recovered children were identified per health post using the one-year therapeutic multi-chart logbook (from November 2016 to October Oct 2017) as a sampling frame. Finally, through these identification procedure, parents were contacted by Health Extension Workers (HEWs) and asked to bring children to the health post for survey data collection, to measure their mid upper arm circumference and to examine them for bilateral oedema according to anthropometric standards.

Children’s vaccination status was checked by looking at their immunization card and if not available, mothers were asked to recall if the child was vaccinated in as much detail as possible especially the last vaccination when they told by health professionals that the child has now completed his/her vaccination to know indirectly for measles vaccination and Bacillus Chalmette Guerin (BCG) vaccination for tuberculosis was also checked if any scar existed on a child’s arm.

### Study variables

The outcome variable was relapse of acute malnutrition after discharged as recovered from the CMAM program in South Gondar Zone, Amhara region, Ethiopia.

Independent variables included socio-economic status, demographic data, household hygiene/sanitation, awareness of recommended caring and feeding practices for children, health facility access, and household food security.

### Data collection tools and measurements

The data collection tools used consisted of a checklist and questionnaire. The checklist was prepared using the stabilization Centre/outpatient therapeutic program (SC/OTP) multi-chart and registration logbook which are utilized throughout the country [13, 28] to identify and trace discharged recovered children. In addition, the questionnaire used for this study was adapted from validated locally used questionnaires in nutrition research and survey reports. For example questions and potential responses regarding socio-demographic/economic, housing conditions, child feeding/caring and related items were developed from the Ethiopian Demographic and Health Survey reports [29].

In addition, the questions to assess HH food security status were taken from a validated questionnaire developed by Food and Nutrition Technical Assistant (FANTA) project [30]. For further validation, prior to data collection, the questionnaires were pre-tested on 5% of actual respondents in another health facility (Health Post) outside of the study area to check for understandability and clarity, and appropriate corrections were made based on the pre-test results.

The questionnaire was prepared in English and then translated to the local language (Amharic) and back to English to check the consistency. Mothers or primary caretakers were interviewed using the questionnaire which took approximately 20–30 minutes. The data collection took place from 10 November 2017 to 30 January 2018. A total of 15 data collectors who had SAM management training and relevant previous experience in data collection were recruited and trained for 2 days, with training content mainly focusing on anthropometric measurement techniques and on how to administer the questionnaire [3, 28]. The data collectors were closely supervised by the 3 trained health professionals and by the principal investigator.

### Anthropometric measurements

Child MUAC was measured halfway between the olecranon and acromion process using non-stretchable tape snugly at the midpoint according to the standard and tape was checked as not pulled too tight or too loose and recorded to the nearest 0.1cm. In addition, presence of edema was assessed by grasping both feet in the hands with the thumbs on top of the feet and then pressing the thumbs gently for three seconds or a count of 101,102,103 and then releasing the thumbs. It was recorded as “0” if no pitting was detected on the feet, recorded as “+” if an indent was detected on feet, “++” if on legs and feet, and “+++” if it included the hands and face according to accepted standards [3, 13].

To assure data quality, data collectors and supervisors were trained for 2 consecutive days on anthropometric measurement techniques [13, 28] and were also closely supervised by supervisors and investigators. Pre-testing took place in a health post from a non-study district similar to the study sites.

### Operational definitions/definition of terms

Relapse: a child was considered as a relapsed case, if he/she had MUAC <12.5cm (both SAM and MAM) and/or presence of bilateral edema after having been successfully discharged recovered from CMAM [3, 13], "Cases therefore included children who had relapsed to MAM or SAM, while controls were those who had maintained MUAC >12.5cm in the previous one year.

A respondent was categorized as having good hand washing practice if they reported washing hands at 3 or more of the recommended critical times/points (before eating, before preparing food, after defecation, and after cleaning child’s bottom).

A household was considered to have improved drinking water if the source was either from a pipe, protected spring, protected well and/or boiled water.

A respondent was considered as currently on family planning if she reported using any family planning methods currently to avoid pregnancy or extend the interval between births.

Household food insecurity status was determined using the 9 item Household Food Insecurity Access Scale (HFIA) question. and Prior to assigning the food insecurity category (access), each frequency of occurrence responses was coded as 0 for all cases where the answer to the corresponding occurrence question was “no” and then the four food security categories were computed and created sequentially as recommended by FANTA [30]. Finally, the HFIA category 1 was considered as food secure and the remaining as food insecure.

### Data management and analysis

The questionnaire was checked manually for completeness and was entered into EpiData version 3.3.2, and exported to SPSS version 20 for analysis. Data cleaning was done using frequency distribution. Anthropometric indices were generated according to the WHO’s 2006 Child Growth Standards [31] using WHO Anthro software 3.2.2.

Bivariate and multivariable binary logistic regression was employed to identify predictors of acute malnutrition after children were discharged as recovered from CMAM program and those attributable variables with P value <0.05 were entered in to the final multivariate binary logistic regression model. Variables in the final model with P value <0.05 were declared statistically significant.

Strength of association was determined using the odds ratio with 95% CIs. The final model was tested for model goodness of fit using Hosmer Lemeshow test. The standard error (SE) values of variables in the final model were checked for multicollinearity. Mean and standard deviation were used for continuous variables, and frequency and percentages for categorical variables when analyzing.

### Ethical review

The protocol and consent form was approved by the institutional review board (IRB) of the College of Health Sciences of Addis Ababa University, with an IRB protocol number of 068/16/SPH and meeting number of 001/2917. Written permission letters were obtained from Regional, Zonal and District Health Offices of Amhara region. Informed verbal consent was obtained from all study respondents after the purpose, risk, benefit, confidentiality, and their degree of involvement were fully explained to parents or caregivers by their local language and then data collectors signed on the consent form to indicate respondent’s agreement to start the interview. Children with MUAC <11.0cm or presence of edema were linked to OTP as this was the currently used admission criteria to therapeutic care. Nutrition education was given to all respondents after interview.

## Results

### Background characteristics

Among 1,290 respondents, 1,273 (445 cases & 828 controls) were interviewed providing a 97.8% overall response rate (100% for cases and 94.7% for the controls). All respondents were female, rural dwellers and Amhara in ethnicity. Respondent mean (±SD) age in years was 29.9 (±6.8) for cases and 28.9 (±6.6) for controls, respectively. Regarding child related variables, the mean age was 23.1 (± 9.1) months for the cases and 23.1(± 9.8) for the controls and also more than half (56% cases and 53% controls) were in the age range of 12–23 months. Nearly 2/3^rd^ (60%) and half (50%) of children from the cases and controls were male children. About 66% and 57% of the respondents were unable to read and write for cases and controls, respectively. About 80% and 84% respectively of the cases and the controls were farmers by occupation. From the total 445 cases, 155 were SAM and the remaining 290 were MAM cases (See Table 1)

**Table 1:** Background characteristics of mothers and children age 6–59 months following recovery from CMAM, in South Gondar Amhara Region, Ethiopia, 20117/18 (n = 1273).

Variables	Cases, # (%)	Controls, # (%)
HH head (male)	416 (93.5)	768 (92.8)
Age of HH head in years (mean± SD)	37.8 (8.4)	36.0 (8.3)
Age of respondent in years (mean ± SD)	29.9 (6.8)	28.9 (6.6)
15–19	15 (3.4)	35 (4.2)
20–29	174 (39.1)	403 (48.7)
30–39	214 (48.1)	314 (37.9)
40+	42 (9.4)	76 (9.2)
Age of children	23.1(9.1)	23.1 (8.9)
6–11	20 (4.5)	30 (3.6)
12–23	250 (56.2)	439 (53.0)
24–35	119 (26.7)	268 (32.4)
36–47	42 (9.4)	67 (8.1)
48–59	14 (3.1)	24 (2.9)
Child sex (Male)	268 (60.2)	412 (49.8)
Place of district		
Ebnat	244 (54.8)	368 (44.4)
Tach-Gayint	171 (38.4)	314(37.9)
Lay-Gayint	30 (6.7)	146 (17.6)
Religion (Orthodox Christians)	407 (91.5)	786 (94.9)
Respondent occupation (farming)	415(93.3)	773(93.4)
Marital status (currently married)	417 (93.7)	768 (92.8)
Respondent education status (unable to read & write)	293 (65.8)	475 (57.4)
Partner education status (unable to read & write)	234 (56.1)	380 (45.9)
Partner occupation (n = 1185) (farming)	402 (96.4)	735 (95.7)
HH family size (> = 5)	268 (60.2)	447 (54.0)
HH under five children (2+)	131 (29.4)	197 (23.8)
Decision maker on expenditure (both husband & wife)	166 (37.3)	346 (41.8)
HH own farm animals	416 (93.5)	752(90.8)
HH own farmland	417 (93.7)	779 (94.1)
Own at least one HH effects (Yes)	233 (52.4)	491(59.3)
HH food secure (yes)	284 (63.8)	668 (80.7)
HH currently on food Aid	136 (30.6)	207 (25.0)
Attend ANC (Yes)	348 (78.2)	673 (81.3)
Place of delivery (Home)	162 (36.4)	293 (35.4)
Consume extra food during pregnancy/lactation (yes)	242 (54.4)	559 (67.5)
Currently on family planning	314 (70.6)	625 (75.5)
*Key*: *HH (household*, *ANC (Antenatal Care)*		

### Child feeding/caring, housing condition & related characteristics

Nearly two third of respondents reportedly (61% for the cases and 66% for the controls) initiated breast feeding immediately within one hour after birth. Prelacteal feeding was practiced more among the cases than the controls (9.4% vs.3.6%). More than half (51% cases and 54% controls) of respondent households’ water source was improved. About 64% of the cases and 81% of the controls were given vitamin A in the 6-month preceding the survey and also 53 to 54% of children both from the cases and controls were discharged recovered within the last 4–6 months, with an average of 5.2 months since exit from CMAM as recovered (see Table 2).

**Table 2:** Child feeding/caring and housing condition of mothers of recovered children age 6–59 months in South Gondar Zone, Amhara region, Ethiopia, 2017/18, n = 1273).

Variables	Cases, # (%)	Controls, # (%)
Timely BF initiation (Within 1 hr. of birth)	272 (61.1)	550 (66.4)
Colostrum given (Yes)	304 (68.3)	639 (77.2)
Practiced prelacteal feeding (Yes)	42 (9.4)	30 (3.6)
Currently on breast feeding (Yes)	370 (83.1)	689 (83.2)
Frequency of BF/day (including night)		
8–12	88 (19.8)	117(14.1)
<8	28 (6.3)	42 (5.1)
As long as child needs	305 (68.5)	623 (75.2)
Don’t remember/don’t know	24 (5.4)	46 (5.6)
Prepare food separately for children from family diet (Yes)	216 (48.5)	543 (65.6)
Trained on child food preparation (Yes)	195 (43.8)	507 (61.2)
HH latrine type (Pit with or without a slab)	379 (85.2)	725 (87.6)
HH water source (Improved)	226 (50.8)	449 (54.2)
Time to water source (< = 15 minute)	59 (13.3)	182 (22.0)
Hand washing practice (Good)	220 (49.4)	469 (56.6)
Often wash hands with (Soap/ash)	182 (40.9)	406 (49.0)
HH dry waste disposal (Open filed)	273 (61.3)	413 (49.9)
Child feces disposal (Open field)	190 (42.7)	369 (44.6)
Duration after recovery in month (Mean ± SD)	5.3 (2.4)	5.1(2.3)
≤3	117 (26.3)	227 (27.4)
4–6	215 (48.3)	425 (51.3
≥7	113 (254)	176 (21.3
Another HH child currently being treated for SAM (Yes)	19 (4.3)	17 (2.1)
De-worming tablet given in last 6 months (Yes)	87 (19.6)	191 (23.1)
Vitamin A supplementation in the past 6 months (Yes)	286 (64.3)	669 (80.8)
Vaccinated for measles (Yes)	420 (94.4)	795 (96.0)
Illness history (Diarrhea, fever, cough) in last 2 wks.	68 (15.3)	129 (15.6)
Treatment stay in week (Mean ± SD)	7.9 (1.9)	8.1 (1.6)
< 8.0	210 (47.2	290 (35.0)
≥8.0	235 (52.8)	538(65.0)

### Factors associated with acute malnutrition following discharge as recovered from CMAM program

Variables were checked whether they were factors associated with post-discharge relapse of acute malnutrition following discharge as recovered from CMAMA program using bivariate logistic regression model and a total of 24 candidate variables with P value <0.05 were identified and entered in to the multivariate regression model to control confounding. Eleven variables retain their statistical significance when entered in to the final regression model.

The model goodness of fit was tested using Hosmer Lemeshow; X^2^ (8, n = 1273) = 10.801, P. value = 0.213, indicating good model fit. In addition, multicollinearity was checked using the standard error (SE) values of variables and were in the range of 0.126 to 0.358 indicating no multicollinearity.

The odds of relapse of acute malnutrition were 1.7 times higher among male children (AOR = 1.75; 95% CI: 1. 36–2. 2.25); 2 times higher among those who had received pre-lacteal feeding (AOR = 2.36; 95% CI:1.38–4.02); 2 times higher in those residing in Ebnat district (AOR = 1.90; 95% CI: 1.19–3.02); 1.5 times higher among children in food insecure HH (AOR = 1.48; 95% CI:1.11–1.99), and almost 2 times higher among children not given vitamin A supplement (AOR = 1.98; 95% CI: 1.47–2.65) see Table 3 below for further detail).

**Table 3:** Factors associated with relapse of relapse of acute malnutrition among children age 6–59 months following discharge as recovered from CMAM in South Gondar Zone Amhara region Ethiopia, 2017/18 (n = 1273).

Variable (final regression)	Response category	Wasting		95% C. I			
		Cases	Controls	Crude OR	P value	Adjusted OR	P value
Child Sex	Male	268	412	1.53 (1.21–1.93)	<0.001	1.75 (1.36–2.25)	0.0001
	Female	177	416	1		1	
Vitamin A supplemented	No/Don't know	159	159	2.34(1.80–3.03)	<0.001	1.98 (1.47–2.65)	0.001
	Yes	286	669	1		1	
HH food security status	Insecure	161	160	2.37 (1.83–3.07)	<0.001	1.48 (1.11–1.99)	0.009
	Secure	284	668	1		1	
Respondent age	> = 30	256	390	1.52 (1.21–1.92)	<0.001	1.41 (1.09–1.83)	0.009
	<30	189	438	1		1	
District of residence	Ebnat	244	368	3.23 (2.11–4.94)	<0.001	1.90 (1.19–3.02)	0.007
	Tach-Gayint	171	314	2.65 (1.72–4.09)	<0.001	1.40 (0.87–2.25)	0.168
	Lay Gayint	30	146	1		1	
Respondent education status	Non formal	397	663	2.06 (1.46–2.91)	<0.001	1.42 (0.96–2.10)	0.075
	Formal	48	165	1		1	
Pre-lacteal fed	Yes	42	30	2.77 (1.71–4.50)	<0.001	2.36(1.38–4.02))	0.002
	No	403	798	1		1	
Colostrum fed	No/not sure	141	189	1.57(1.21–2.03)	0.001	1.25 (0.93–1.68)	0.141
	Yes	304	639	1		1	
Religion	Muslim	38	42	1.75 (1.11–2.75)	0.015	1.44 (0.85–2.46)	0.180
	Orthodox	407	786	1		1	
Trained in child food preparation	No	250	321	2.03 (1.60–2.56)	0001	1.16 (0.85–1.58)	0.343
	Yes	195	507	1		1	
Hand washing practices	Poor	225	359	1.34 (1.06–1.68)	0.014	1.33 (1.04–1.70)	0.024
	Good	220	469	1		1	
HH dry waste disposal	Open field/bush	273	413	1.60 (1.26–20.2)	<0.001	1.15 (0.85–1.55)	0.370
	Put pit/burn	172	415	1		1	
Additional fed during pregnancy/ lactation	No	203	269	1.74(1.38–2.21)	<0.001	1.35 (1.04–1.75)	0.026
	Yes	242	559	1		1	
Another HH child currently on SAM therapy	Yes	19	17	2.13 (1.09–4.14)	0.023	2.16(1.07–4.35)	0.032
	No	426	811	1		1	
Time to drinking water source in minute walk	>15/don’t know	386	646	1.84 (1.34–2.54)	<0.001	1.72 (1.22–2.42)	0.002
	≤15	59	182	1		1	
Treatment stay in weeks	<8	210	290	1.66 (1.31–2.10	<0.001	1.44 (1.12–1.86)	0.005
	≥8	235	538	1		1	
Own at least one HH effects	No	212	336	1.33 (1.06–1.68)	0.015	1.01 (0.77–1.33)	0.941
	Yes	233	492	1		1	
HH family size	≥5	268	447	1.29 (1.02–1.63)	0.033	1.04 (0.76–1.42)	0.818
	<5	177	381	1		1	
Often wash hands with	Water only	263	422	1.39 (1.10–1.76)	0.006	1.04 (0.77–1.40)	0.808
	Soap/ash	182	406	1		1	
HH currently on food aid	Yes	136	207	1.32 (1.02–1.71)	0.033	1.05 (0.77–1.43)	0.778
	No	309	621	1		1	
Prepare food to child separately from family diet	No	229	285	2.02 (1.60–2.55)	<0.001	0.94 (0.63–1.41)	0.775
	Yes	216	543	1		1	
HH head age in yrs.	≥36	235	364	1.43 (1.13–1.80)	0.003	0.91 (0.63–1.30)	0.597
	<36	210	464	1		1	
HH Under five children	2 or more	131	197	1.34 (1.03–1.73)	0.028	1.19 (0.89–1.59)	0.239
	1	314	631	1		1	

## Discussion

In the current study, children were assessed after an average of 5.2 months of recovery and ten variables were identified as being associated with relapse of acute malnutrition (child sex, district of residence, HH food security status, pre-lacteal feeding, Vitamin A supplementation, consumption of extra food during this pregnancy/lactation, time to water source, another HH child on SAM therapy, frequency of hand washing and age of respondents in years)

The odds of relapse of acute malnutrition were higher among male than female children. This finding was in line with studies conducted in rural Southern Malawi [21], rural Malawi [32], in Sierra Leone [33] and in Zambia [34]. One possible reason for this disparity could be the typical activities of boys in the region to be away from the home performing tasks related to pastoral agriculture, leading potentially to missed meals. A recent study has also noted that boys health may be more influenced by environmental stressor and diarrhea [35].

The odds of relapse were higher among children not given Vitamin A in the 6 months preceding the survey than among those who received Vitamin A supplement. This finding may indicate low receipt of, or access to nutrition services, such as distribution of Vitamin A capsules and was in agreement with a studies conducted in Hawassa, and Afar region, Ethiopia [36, 37]. This could also potentially reflect the role of Vitamin A in promoting and regulating activities in both the innate and adaptive immune system [38], therefore enhancing immune function [39] and is in line with the rationale for recommended supplementation every 6 months [8, 40]. In addition, keeping high risk children in the CMAM program for a longer period and also linking them to supplementary feeding program could also help in reducing relapse [21, 32, 41].

In the current study, children who were given pre-lacteal feeding were more acutely malnourished than those who were not. This finding affirms those in other studies conducted in India and in Dollo Ado district of Somali Region Ethiopia reporting that families which practiced pre-lacteal feeding had more acutely malnourished children [42–44]. This finding relates to the introduction of pre-lacteal feeds which disrupts the feeding of colostrum, practice of EBF, and the increases likelihood of other foods being introduced before 6 months as has been reported in Amhara Region Ethiopia [45, 46]. This practice is likely to result in enteric infections and environmental enteropathy due to consuming of unsafe water or liquids, emphasizing the need to counsel families on prompt initiation and exclusive breastfeeding [47], while supporting changing norms, as well as provision of appropriate counseling not to provide water [48] due to risks associated [49].

In the current study, place of residence (district) was associated with relapse of acute malnutrition. Topographically, Lay Gayint is one of the highland and mountainous areas in the Zone, while Ebnat is more lowland but both districts are drought-prone and rural. Children who lived in Ebnat district were more acutely malnourished compared to those who lived in Lay Gayint district. This could be due to the difference in the exposure to infectious diseases such as malaria as it is common in lowland than in highland area and children infected with malaria were found more acutely malnourished than non-exposed children [50]. This could be related also to differences in HH food security status, hand washing practices, and access to water source in the districts. In the current study, more than half (54.7%) of food insecure HHs, of which 87.5% (49/56) were considered severely insecure were located in Ebnat, and nearly half (47.3%) of respondents who had poor hand washing practices and 48.8% of Households whose water source was longer than a 15-minute walk were at Ebnat district. Geographic variation has also been noted in Bangladesh stating that more acutely malnourished children were located in the Eastern versus the Southern district [51], as well as in Sylhet versus Dhaka division [52], and in Northern provinces of Zambia versus in the Western provinces [34].

As might be expected relapse of acute malnutrition after discharge as recovered was also related to HH food security status generally. The odds of relapse among children in food insecure HHs was higher than in food secure HHs. The finding was in agreement with studies conducted in India [25], Nigeria [53] and Ethiopia [36]. Food insecurity can be linked to inadequate intake of diversified foods and studies have reported consumption of low dietary diversity food as being associated with acute malnutrition [54, 55]. Other studies pointed to the role of low socioeconomic status or monthly income in food insecurity [56–59] which directly or indirectly reduces the HH purchasing power, and thus, reduces access to food.

Having another family member currently on SAM therapy increased the likelihood of being more acutely malnourished. In the current study, children in food-insecure households were more acutely malnourished than their counterparts, a finding supported by results of other recent studies [25, 53]. Low HH socioeconomic status could contribute to children to being acutely malnourished [56, 57] which in turn may lead HHs to feed children less diversified diets [54, 55]. In the current study, 1.1% of respondents reported sharing therapeutic food with other children in the family and1.2% reported knowing other households which had done so. Sharing therapeutic food could make it more likely that a recovered malnourished child could relapse. As reported by Concern Worldwide, some children at feeding centers have been kept in the programme longer than the required duration due to therapeutic food sharing with other children [60]. Also in India, it was reported that although Balbhog (Energy Dense Micronutrient Fortified Extruded Blended Food) was provided as supplementary nutrition, it was also shared with other family members [61].

The odds of relapse were higher among children whose mother reported not consuming additional food during this pregnancy/lactation. International recommendations for pregnant and lactating women include additional daily food consumption to meet extra caloric needs [62]. Our finding echoes research indicating that undernourished (BMI<18.5) mothers are more likely to have undernourished children [51, 57, 63–65]. Finding have also been reported that pregnant mothers with high dietary diversity scores have improved gestational weight gain [66].

In the current study, children in a HH with access to drinking water within 15-minutes walking distance were less acutely malnourished than those whose access to drinking water was greater than 15-minutes walking distance or more. This finding is similar to that of a study done in Dollo Ado district, Somali region, Ethiopia [42]. Those with a shorter walking distance were less acutely malnourished compared to those with a walking distance of greater than 60 minutes [67]. As reported by Pickering and Davis, if the water source is far from home, then mothers will need to spend significantly more time fetching water, and thereby have less time to care for children [68]. This association could also be linked with the well- known relationship between better water, sanitation and hygiene access with improved nutrition outcomes [69].

The World Health Organization (WHO) recommends that for optimal health outcomes families should wash hands at four key time points/critical periods: before eating, before preparing food, after defecation, and after disposal of child feces to prevent infection and malnutrition [69]. In the current study, the odds of acute malnutrition were higher among children whose mothers had practiced hand washing only after one or two of the key points compared to those who wash hands frequently (three or more of the hand washing time points). These findings of a relationship between adherence to hand washing practices are similar to those from studies in Bangladesh, Chad, and Ethiopia where caregivers who washed hands regularly were less likely to have acutely malnourished children [55, 70–72]. Per the principle of infection malnutrition cycle, if a mother washes her hands at the critical points, then the risk of contamination by excreta and the transmission of the pathogens is prevented, along with nutritional deficiencies [73].

Age of the index child’s mother was also a factor associated with post-discharge relapse, with children whose mother was above 30 years were being more likely to be malnourished. A study in Nepal also identified an association between acutely malnourished children and mothers aged above 35 years [74]. This could be due to the fact that as the age of the mother increases, the number of pregnancies could increase and, if the spacing between pregnancies was close; her nutritional resources are more depleted. This link was supported by a recent study conducted in Ethiopia stating that the risk of children being acutely malnourished decreases as birth intervals increases (> = 24months) [75].

According to the current CMAM protocol, the length of treatment stay at OTP is expected to be <8 weeks, otherwise considered as poor performance [13] but in the current study, children who were discharged as recovered within 8 weeks of admission to OTP were found more acutely malnourished compared with children discharged after 8 weeks. This could be because, they were discharged as long as they achieve 15% weight gain [13] but may not be fully recovered. A study in Sudan indicated the disadvantage of using 15% target weight gain discharge criteria compared with MUAC describing the benefit that use of MUAC ≥ 125mm as discharge criteria gave children longer duration of treatment stay and a higher percent weight gain [76]. A study in Southern Ethiopia has indicated that 9% of discharge as recovered children based on 15% target weight gain were still moderately or severely wasted [77]. Another study based on 15% weight gain discharge criteria also reported a high prevalence of SAM (34.6%), 14 weeks following discharge [22].

### Strengths and limitations

The study is the first study among children successfully discharged recovered from CMAM in the study area to identify key associated factors with post-discharge relapse of acute malnutrition; such information will help program managers and nutrition focal persons to address acute malnutrition. This study however has limitations: The cases and controls were identified using MUAC only and not using both MUAC and WHZ as the two indicators could identify relapsed cases differently. The factors associated with relapse of acute malnutrition “provision of colostrum, Vitamin A supplementation, and distance from water sources” need to be interpreted cautiously as the responses “don’t know, not sure, and/or don’t remember” were classified as “no” assuming that children were not given colostrum, Vitamin A, and/or distance to water source was more than a 15minute walk. Food insecurity status could be underestimated as the data collection time was in the harvest season (October 2017 to January 2018).

## Conclusion and recommendation

Several risk factors were associated with relapse of acute malnutrition following recovery (sex of the child, place of residence, food security status, pre-lacteal feeding, caretaker hand washing practices, receipt of Vitamin A supplementation, household access to water source, maternal consumption of additional food during pregnancy/lactation, age of the mother, length of treatment stay, and presence of another child in the household receiving nutritional therapy). Understanding these factors may lead to the ability to predict and prevent future post discharge relapse of acute malnutrition. WASH and prelacteal feeding practices were among the drivers of optimizing child nutrition conditions following recovery, therefore, improvements in caregiving practice with mothers being the critical entry point for behavior change should be one priority interventions along with CMAM program. In addition, household food insecurity interventions through public Safety Net programs should be strengthened.

## Supporting information

S1 Data (SAV)
