# Correction: Predictors of relapse of acute malnutrition following exit from community-based management program in Amhara region, Northwest Ethiopia: An unmatched case-control study

**DOI:** 10.1371/journal.pone.0294299

**Published:** 2023-11-07

**Authors:** Dereje Birhanu Abitew, Alemayehu Worku Yalew, Afework Mulugeta Bezabih, Alessandra N. Bazzano

An additional affiliation is missing for the first author. Dereje Birhanu Abitew is also affiliated with: School of Public Health, Bahir Dar University, Addis Ababa, Ethiopia.
